# Pathological Laughter as a Symptom of Midbrain Infarction

**DOI:** 10.1155/2004/409248

**Published:** 2005-01-27

**Authors:** Ron Dabby, Nathan Watemberg, Yair Lampl, Anda Eilam, Abraham Rapaport, Menachem Sadeh

**Affiliations:** ^1^Department of NeurologyEdith Wolfson Medical CenterHolon 58100Israel Affiliated to Sackler Faculty of MedicineTel Aviv UniversityTel AvivIsrael; ^2^Pediatric Neurology UnitEdith Wolfson Medical CenterHolon 58100Israel Affiliated to Sackler Faculty of MedicineTel Aviv UniversityTel AvivIsrael

## Abstract

Pathological laughter is an uncommon symptom usually caused by bilateral, diffuse cerebral lesions. It has rarely been reported in association with isolated cerebral lesions. Midbrain involvement causing pathological laughter is extremely unusual. We describe three patients who developed pathological laughter after midbrain and pontine-midbrain infarction. In two patients a small infarction in the left paramedian midbrain was detected, whereas the third one sustained a massive bilateral pontine infarction extending to the midbrain. Laughter heralded stroke by one day in one patient and occurred as a delayed phenomenon three months after stroke in another. Pathological laughter ceased within a few days in two patients and was still present at a two year follow-up in the patient with delayed-onset laughter. Pathological laughter can herald midbrain infarction or follow stroke either shortly after onset of symptoms or as a delayed phenomenon. Furthermore, small unilateral midbrain infarctions can cause this rare complication.

## 1. Introduction

Laughter is defined as pathological when it is inappropriate, uncontrollable or continuous. Pathological laughter (PL) is commonly associated with bilateral, multiple or diffuse cerebral lesions. In such instances, it is usually accompanied by crying as a part of a pseudobulbar syndrome [1]. PL rarely results from an isolated brainstem lesion. In recent years a few reports describing PL in association with a focal lesion involving the deep white or gray matter have been published [2, 3]. Reports of isolated brainstem lesions causing PL are anecdotal [2,4–6]. We hereby describe three patients who presented with PL, and in whom neuroimaging studies demonstrated an isolated brainstem lesion.

## 2. Case reports

### *2.1. Patient 1*

A 49-year-old man suddenly developed involuntary and inappropriate laughter. He experienced short episodes of uncontrollable laughter lasting several seconds and occurring either spontaneously or during conversation. He had no other symptoms at onset. One day after PL started, he developed right hemiparesis and dysarthria. His previous medical history was significant for non-insulin-dependent diabetes mellitus, hypertension, and hyperlipidemia. On examination he appeared alert and had many bursts of inappropriate laughter. There was moderate right hemiparesis with weakness of the right facial muscles. Deep tendon reflexes were brisk on the right with ipsilateral extensor plantar response. Sensory and cerebellar functions were intact. The laughter gradually subsided over a week. The dysarthria improved, but not the hemiparesis. Brain CT one day after onset of PL was normal. Repeat CT scan five days later showed an ischemic in-

ISSN 0953-4180/04/$17.00 © 2004 – IOS Press and the authors. All rights reserved

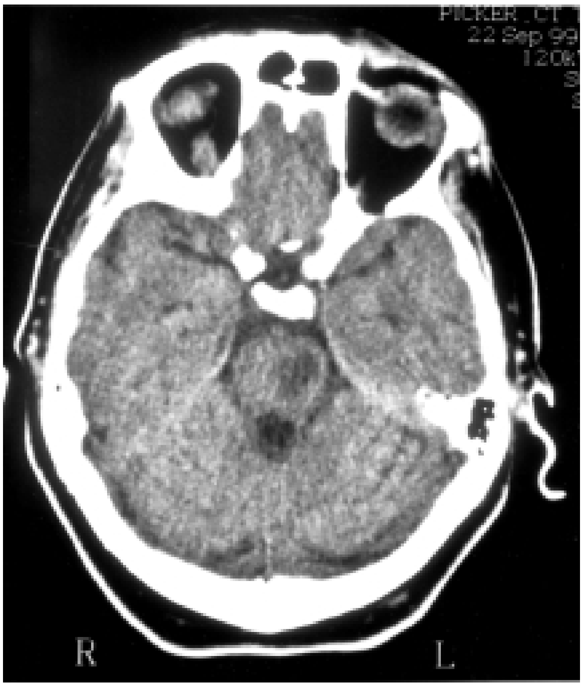

Fig. 1. Patient 1. CT five days after onset of symptoms showing ischemic infarction in the lower left midbrain.

farction at the paramedian area of the lower midbrain on the left (Fig. 1).

### *2.2. Patient 2*

A 59-year-old man with hypertension and hypercholesterolemia had been on coumadin therapy for five years following pulmonary embolism. At age 57 he was diagnosed with a vertebrobasilar stroke, which manifested with vertigo, loss of consciousness, and brief right hemiparesis. Brain CT, transesophageal echo (TEE), cardiac Holter, and chest X- ray were normal. Carotid duplex showed minimal atheromatotic plaques on both sides. Laboratory work-up was normal, except for a heterozygous state for APCR. He recovered completely and resumed a full and active life-style. Three months after the stroke involuntary laughter appeared. It was uncontrollable, inappropriate, and occurred in short bursts, either spontaneously, during conversation, or in response to trivial stimuli. Brain MRI four months after the event (one month after the onset of PL) showed a small ischemic lesion in the paramedian area of the lower midbrain on the left. There was no evidence of ischemia elsewhere. PL was still present at a two year follow-up exam.

### *2.3. Patient 3*

A 64-year-old woman was admitted because of vertigo and right internuclear ophthalmoplegia. She suffered from hyperlipidemia, peptic ulcer, and rheumatoid arthritis. Five days after onset of symptoms left hemiparesis appeared and bouts of inappropriate uncontrollable laughter ensued. PL occurred either spontaneously or was provoked by trivial stimuli. Over a period of several days she deteriorated into a locked-in state, and shortly thereafter became comatose and was intubated. Overall, laughter lasted several days and ceased when she developed locked-in syndrome. Brain CT scan at onset was normal. Repeat CT scan 10 days later showed a massive bilateral ischemic infarction at the base of the pons extending to the lower midbrain.

## 3. Discussion

Pathological or inappropriate laughter is an uncommon occurrence. It may be associated with diffuse CNS pathologies such as multiple sclerosis [7] and amyotrophic lateral sclerosis [8]. Gelastic seizures are rare epileptic events characterized by uncontrollable bouts of laughter, usually but not always caused by hypothalamic hammartomas [9]. Moreover, intravenous valproate administration was followed by PL in two patients [10]. In cases of bilateral or diffuse cerebral lesions, particularly of ischemic or degenerative nature, PL occurs as part of a pseudobulbar syndrome, which often involves inappropriate crying too [1,11]. In these cases it is frequently difficult to determine if PL is related to the brain lesion or whether inappropriate laughing occurs as a manifestation of emotional instability or depression [2]. PL may also be a manifestation of trigeminal nerve and trochlear nerve neurinomas [12, 13]. PL occurring at the onset of cerebrovascular disease has been termed “fou rir e prodromique” (prodromic laughter) [14].

PL as a result of an isolated cerebral lesion has uncommonly been described. Most reported patients have had subcortical lesions in areas such as the internal capsule, thalamus, basal ganglia and the deep white matter [2,3,15]. Unlike the case of pseudobulbar syndrome, isolated lesions may cause inappropriate laughter without concomitant crying [3,14].

Reports of isolated brainstem lesions causing PL are scarce. A brainstem infarct occurred in only three of 13 patients with unilateral stroke who developed PL [2]. In these three patients PL started acutely or subacutely following the stroke. A large series of patients with poststroke depression or postsroke emotional instability (including PL) found that these conditions could be produced by ischemic or hemorrhagic brain lesions in the frontal lobes, temporal lobes, occipital lobes, brainstem, lenticulocapsular area, and the cerebellum [16]. Among brainstem lesions the basis pontis and the medulla were mostly associated with emotional incontinence. The authors conclude that poststroke depression and poststroke emotional instability are likely to arise from chemical neuroanatomical changes in the frontal/temporal lobe-basal ganglia-ventral brainstem circuitry. Interestingly, two of our patients had an isolated midbrain lesion. In the third case both the pons and midbrain were involved.

In our series PL preceded the symptoms of stroke by 24 hours in one case. It appeared five days after the ischemic event in the second patient, and in the third patient it occurred three months after the stroke. PL sometimes precedes the motor signs of stroke [3,8,17] or it may be the sole manifestation of a brainstem hemorrhage [6]. However, in the largest series of unilateral stroke-associated PL, it occurred days to up to one year after stroke occurrence. Although the number of cases in that series was rather small, Kim detected a trend for large subcortical strokes to correlate with delayed onset PL, presumably due to cortical dysfunction. On the other hand, laughter occurred relatively early in patients with small subcortical lesions or pontine base infarcts that were unlikely to have produced cortical dysfunction [2]. Of note, one of our patients had delayed onset PL despite having a small midbrain infarct.

The pathogenetic mechanism of PL is not yet clear. Normal laughter is probably controlled by a neuroanatomic circuitry, given the multiple conditions and the varied anatomical locations of brain lesions in patients with PL. This circuitry probably includes: the anterior cingulate gyrus, involved in the expression of emotion and laughter; the amygdala which provides the cingulate gyrus with the emotional coloring of perceptions; the parahippocampal and fusiform cortices in the temporal lobe that integrate perceptions with previous experiences; the caudal hypothalamus which coordinates internal emotional changes; and the ventral pontomedullary center for laughter which coordinates emotional vocalization, facial expression, and expirations. In addition, bilateral corticobulbar tracts tonically inhibit laughter and probably suppress the ventral pontomedullary laughter center [18]. A supranuclear pontobulbar center controlling input to the motor nuclei of the brain stem and the anterior horn cells in the upper cervical cord has been suggested [17]. Poeck speculated that there is tonic and phasic innervation of the movement of facial expression and that in "fou rire prodromique" there is loss of onic control of laughter [1].

It is possible that the brainstem lesion interrupted the presumed laughter-control pathways, thus leading to PL in our patients. The occurrence of PL in one patient several months after the stroke suggests a more complex mechanism rather than a simple disruption of the laughing-related pathways. Presumably, delayed-onset PL reflects the time required for the formation of autonomous motor centers. This may be equivalent to the delayed-onset hyperkinetic movements or palatal myoclonus developing after a focal cerebral lesion. It is of interest that PL lasted for many months in the patient in whom it started long after the stroke. This supports the hypothesis for the formation of new neuronal activity. Hence, there might be two mechanisms leading to PL: One would be immediate and probably related to disruption of a brain stem-basal ganglia-forebrain circuitry, and the other one would be delayed, presumably due to development of new neuronal activity or new laughter-control centers.

It appears that serotonergic neurotransmission may be damaged in patients with emotional instability and PL. Indeed, serotonin reuptake inhibitors (SSRIs) can markedly improve this condition in cases of pseudobulbar palsy [19,20]. There are no reports, however, on the use of SSRIs in PL associated with brainstem lesions.

Our study suggests that single midbrain lesions may also play a role in the pathogenesis of PL. In the patient that sustained a massive bilateral pontine-midbrain infarction it is not possible to determine the exact site responsible for PL because of the large size of the lesion. The occurrence of PL as a heralding symptom of stroke harbours important therapeutic implications, since early intervention before the appearance of other neurological symptoms may be crucial. More studies are required to clarify the pathogenesis of post-stroke PL and its prevalence.
